# Chronic Exposure to GLP-1 Increases GLP-1 Synthesis and Release in a Pancreatic Alpha Cell Line (α-TC1): Evidence of a Direct Effect of GLP-1 on Pancreatic Alpha Cells

**DOI:** 10.1371/journal.pone.0090093

**Published:** 2014-02-28

**Authors:** Salvatore Piro, Loriana G. Mascali, Francesca Urbano, Agnese Filippello, Roberta Malaguarnera, Salvatore Calanna, Agata M. Rabuazzo, Francesco Purrello

**Affiliations:** 1 Department of Clinical and Molecular BioMedicine, University of Catania, Garibaldi-Nesima Hospital, Catania, Italy; 2 Endocrinology, Department of Health Sciences, University Magna Graecia of Catanzaro, Catanzaro, Italy; University of Bari Aldo Moro, Italy

## Abstract

**Aims/Hypothesis:**

Incretin therapies, which are used to treat diabetic patients, cause a chronic supra-physiological increase in GLP-1 circulating levels. It is still unclear how the resulting high hormone concentrations may affect pancreatic alpha cells. The present study was designed to investigate the effects of chronic exposure to high GLP-1 levels on a cultured pancreatic alpha cell line.

**Methods:**

α-TC1-6 cell line was cultured in the presence or absence of GLP-1 (100 nmol/l) for up to 72 h. In our model GLP-1 receptor (GLP-1R) was measured. After the cells were exposed to GLP-1 the levels of glucagon secretion were measured. Because GLP-1 acts on intracellular cAMP production, the function of GLP-1R was studied. We also investigated the effects of chronic GLP-1 exposure on the cAMP/MAPK pathway, Pax6 levels, the expression of prohormone convertases (PCs), glucagon gene (*Gcg*) and protein expression, glucagon and GLP-1 production.

**Results:**

In our model, we were able to detect GLP-1R. After GLP-1 exposure we found a reduction in glucagon secretion. During further investigation of the function of GLP-1R, we found an activation of the cAMP/MAPK/Pax6 pathway and an increase of *Gcg* gene and protein expression. Furthermore we observed a significant increase in PC1/3 protein expression, GLP-1 intracellular content and GLP-1 secretion.

**Conclusions/Interpretation:**

Our data indicate that the chronic exposure of pancreatic alpha cells to GLP-1 increases the ability of these cells to produce and release GLP-1. This phenomenon occurs through the stimulation of the transcription factor Pax6 and the increased expression of the protein convertase PC1/3.

## Introduction

Type 2 diabetes mellitus (T2DM) affects millions of people throughout the world [1]. The pathogenesis of this disease involves reduced insulin sensitivity of the targets of insulin action in peripheral tissues, impaired insulin secretion by pancreatic beta cells and altered glucagon secretion by pancreatic alpha cells [2]. In recent years, a new class of drugs has been introduced for the treatment of T2DM. This class of drugs is based on the ability of Glucagon-Like Peptide-1 (GLP-1), a hormone produced by intestinal L cells, to reduce plasma glucose levels in the peripheral tissues. GLP-1 acts at multiple levels but mainly affects pancreatic beta and alpha cells [3]. GLP-1 potentiates the glucose-induced release of insulin and prevents the occurrence of unregulated high glucagon levels often observed in diabetic subjects [4]. Because GLP-1 is rapidly degraded by the enzyme Di-Peptidyl Peptidase Type IV (DPP-4) and therefore has a very short plasma half-life, analogues of GLP-1 that are more resistant to DPP-4 degradation or DPP-4 inhibitors are currently used to treat T2DM [5]. Currently, most GLP-1 analogues and DPP-4 inhibitors are taken once a day; however, preparations with a longer half-life will be soon available. Therefore, an increasing number of diabetic patients are treated with these drugs and are thus chronically exposed to high GLP-1 concentrations (pharmacological levels); due to the reversible binding to plasma proteins, levels of some of these analogues might increase over the time [6]-[8].

The present study was designed to investigate the effects of chronic exposure to high GLP-1 levels (as experienced by T2DM patients treated with GLP-1 analogues or DPP-4-inhibitors) on cultured pancreatic alpha cells (α-TC1 clone 6). The inhibitory effect on glucagon secretion of GLP-1 on pancreatic alpha cells has been described both *in vitro* and *in vivo* [4], [9]. However, it is still unclear whether the inhibition of glucagon secretion mediated by GLP-1 is due to a paracrine (indirect) effect on alpha cells, the stimulation of insulin and somatostatin secretion from neighboring beta and delta cells or a direct effect that occurs through the GLP-1 receptors of alpha cells [10]–[12]. In some previous works it has been shown not only that GLP-1 receptors are present in alpha cells, although at very low levels if compared with beta cells, but also that the inhibition of glucagon release induced by GLP-1 is PKA dependent and does not involve paracrine effects mediated by insulin or somatostatin [13], [14]; however to date this aspect remains still controversial. In our model we first measured the expression of the GLP-1 receptor (GLP-1R) on the surface of α-TC1 cells. Next, we investigated the biological effects of this molecule, including the increase in intracellular cyclic adenosine monophosphate (cAMP) levels, the activation of the MAPK pathway and the inhibition of glucagon release. Subsequently, we investigated the expression of Pax6 and glucagon (*Gcg*) genes and proteins that are known to be activated by the MAPK pathway. Finally, in light of recent data indicating that pancreatic alpha cells are able to synthesize and secrete GLP-1 under certain conditions [15], we evaluated the products of *Gcg* gene (in particular glucagon and GLP-1) and the enzymes involved in proglucagon conversion (specifically, the protein convertases PC2 and PC1/3). These molecules are selected members of a family of subtilisin-like endoproteases known as prohormone convertases (PCs) that generate glucagon and GLP-1 from *Gcg* genes [16].

## Research Design and Methods

### Chemicals and reagents

Cell culture media, active human GLP-1 [7–37 fragment], Exendin-4, Exendin-9 [fragment 9-39], aprotinin from bovine lung and all chemicals, unless otherwise stated, were obtained from Sigma Chemical (Sigma-Aldrich, St. Louis, MO, U.S.A.). Sources for other reagents were as follows: KH7 (Cayman Chemical, Nashville, Tennessee, U.S.A.), Fetal Bovine Serum FBS and Alexa Fluor-549 anti-Rabbit IgG secondary antibody (Invitrogen Laboratories, Carlsbad, CA), anti GLP-1R, anti actin, anti PC1/3 (pcsk1), anti PC2, anti proglucagon (Santa Cruz Biotechnology, Inc., Santa Cruz, CA), anti phospho ERK 44/42 (phospho-44/42 MAPK) (Thr202/Tyr204) and anti-paired box gene 6 (Pax6) (R&D Systems, Minneapolis, MN).

### α-TC1 cell line and cell culture conditions

α-TC1 (clone 6), purchased from the American Type Culture Collection (ATCC, U.S.A., through LGC Standards S.r.l., Milan, Italy), is a pancreatic α-cell line cloned from the **α**-TC1 cell line. This line was derived from an adenoma created in transgenic mice expressing the SV40 large T antigen oncogene under the control of the rat pre-proglucagon promoter. Although the parental **α**-TC1 cell line produces glucagon and considerable quantities of insulin and pre-proinsulin mRNA, the clonal line (clone 6) is terminally differentiated and produces glucagon but not insulin or pre-proinsulin mRNA. **α**-TC1 clone 6 cells exhibit the most differentiated phenotype and express the highest levels of glucagon. These cells therefore possess an advantage over primary islets (as they represent a homogeneous cellular population) and have been previously used to study glucagon secretion and gene expression [17]-[19]. In our laboratory, we measured insulin secretion in these cells but did not find any detectable insulin (data not shown). Cells were grown in DMEM (Dulbecco's Modified Eagle Medium) with 4 mmol/l-glutamine (modified to contain 16.7 mmol/l glucose and 1.5 g/L sodium bicarbonate) supplemented with 10% heat-inactivated dialyzed foetal bovine serum, 15 mmol/l HEPES, 0.1 mmol/l nonessential amino acids and 0.02% BSA under an atmosphere of 95% humidified air-5% CO_2_ at 37°C. The cells were passaged once a week and the medium was replaced twice weekly. Most of the experiments were conducted with cells at passages ranging from 20 to 25, however the response of cells from passages 15 to 35 was similar.

Several studies have been performed in InR1G9 cells, another pancreatic α-cell line producing glucagon. This line was obtained from hamsters and lacks the GLP-1 receptor; therefore, the cell line also lacks receptor function [20]–[23]. InR1G9 cells were cultured in 11 mmol/l glucose RPMI-1640 medium supplemented with 5% FBS, 100 U/ml of penicillin, 100 µg/ml of streptomycin and 2 mmol/l-glutamine under an atmosphere of 95% humidified air-5% CO_2_ at 37°C.

Some other experiments have been performed in rat pancreatic islet (kindly provided by Professor Decio L. Eizirik, Laboratory of Experimental Medicine, Universite Libre de Bruxelles, Brussels, Belgium), isolated by collagenase digestion from male Wistar rats (CharlesRiver Laboratories, Brussels, Belgium), treated following the guidelines of the Belgian Regulations for Animal Care and with approval from the local Ethical Committee [24], [25]. Rat islet (n = 50 for each experimental group) were cultured in Ham's F-10 medium containing 10 mM glucose, 2 mM glutamine, 50 mM 3-isobutyl-L-methylxanthine, 0.5% fatty acid-free bovine serum albumin (BSA) (Roche, Indianapolis, IN, USA), 5% FBS, 50 units/ml penicillin and 50 mg/ml streptomycin.

### Choice of the dose of GLP-1 used

Before starting the experiment, to choose the dose of GLP-1, we performed some GLP-1 dose response experiments, measuring cAMP production (see results). We have chosen the concentration of 100 nmol/l, corresponding approximately to 5–10 times more than the range detected in human or in animals treated with “incretin therapies” [6]–[8], [26], [27]. In fact it is well known that *in vitro* we need a higher drug concentration and a shorter time-frame of exposure than *in vivo* to achieve similar effects.

### Chronic exposure to GLP-1

Twenty-four hours after planting, the α-TC1-6 cells were cultured for 72 hours at 37°C in complete DMEM medium in the presence or absence of GLP-1 (100 nmol/l). GLP-1 was replaced every 12 hours, as previously described [26]. To assess the effect of insulin, the cells were serum-starved for 24 h in medium with BSA 0.1% instead of FBS before stimulation with insulin. Acute stimulation with 10^−9^ M insulin was performed for five minutes, as previously described in a study of glucagon secretion in α-TC1-6 cells [19].

### Measurement of cAMP levels

We measured intracellular cAMP levels to test the downstream biological effects of GLP-1. To determine the acute effects of GLP-1, the cells were grown in the absence of GLP-1 for 72 hours, washed twice and exposed to GLP-1 (100 nmol/l) for two hours at high glucose level (25 mmol/L); subsequently, intracellular cAMP levels were measured. To investigate the chronic effect of GLP-1, the cells were incubated for 72 hours in the presence or absence of GLP-1. Additionally, during the last 12 hours, some groups of both control and GLP-1 pre-exposed cells, were cultured in the presence or absence of GLP-1 (100 nmol/l), Exendin-9 (100 nmol/l), a potent GLP-1 receptor antagonist [28], KH7 (25 µmol/l), a selective inhibitor of soluble adenylyl cyclase [29], or Forskolin (50 nmol/l), a direct cAMP inductor [30], alone or in combination. The cells were then washed twice and incubated for two hours in Krebs-Ringer buffer (KRB) containing 25 mmol/l glucose and 0.5% BSA (pH 7.4) in the presence or absence of GLP-1 (100 nmol/l), Exendin-9 (100 nmol/l), KH7 (25 µmol/l) or Forskolin (50 nmol/l).

### cAMP assay

The cells were lysed in 0.1 M HCl solution at 4°C. The samples were collected in vials and centrifuged at ≥600× *g* at room temperature. The cAMP concentrations in the supernatants were determined with a direct enzyme immunoassay kit (cAMP enzyme immunoassay kit, Direct; Sigma-Aldrich, St. Louis, MO) according to the manufacturer's instructions.

### Glucagon secretion

To investigate the effect of GLP-1 on glucagon secretion in our system, we measured glucagon levels after both acute and chronic GLP-1 exposure. To analyse the acute effects of GLP-1, cells were grown in the absence of GLP-1 for 72 hours, washed and exposed to GLP-1 (100 nmol/l) for two hours; to investigate the chronic effects of GLP-1 exposure, twenty-four hours after planting, the cells were grown in the presence or absence of GLP-1 (100 nmol/l) for 72 hours. At the end of culture the cells were washed and incubated for two more hours in Krebs-Ringer buffer (KRB) containing 25 mmol/l glucose and 0.5% BSA (pH 7.4) in the presence or absence of GLP-1 (100 nmol/l). In some experiments an acute stimulation with 10^−9^ M insulin was performed for five minutes. The samples were collected in vials containing aprotinin (0.1 mg/ml) and kept frozen at −20°C until the glucagon RIA (Radioimmunoassay) analysis. This procedure was carried out using the Glucagon RIA Kit (Millipore Corp. Billerica, MA, U.S.A.) [31] according to the manufacturer's instructions.

### GLP-1 secretion

The cells were grown in the presence or absence of GLP-1 (100 nmol/l) for 72 hours and then incubated for two more hours in Krebs-Ringer buffer (KRB) containing 16.7 mmol/l glucose and 0.5% BSA (pH 7.4) in the absence of GLP-1 (100 nmol/l). The medium samples were then collected in vials containing aprotinin (0.1 mg/ml) and kept frozen at −20°C until the active GLP-1 ELISA analysis (Millipore Corp. Billerica, MA, U.S.A.) [32]. Secretion was normalized to the protein content. Some GLP-1 secretion experiments were performed on rat pancreatic islets cultured up to 72 hours in the presence or absence of Exendin-4 (100 nmol/l) a specific GLP-1 analogue. At the end of culture the islets were washed and incubated for two more hours in Krebs-Ringer buffer (KRB) 0.5% BSA (pH 7.4) in the presence or absence of Exendin-4 (100 nmol/l). All the secretion experiments were performed by incubating the cells in Krebs-Ringer Buffer containing 0,1% DiprotinA (Ile-Pro-Ile, Sigma-Aldrich) a specific dipeptidyl peptidase IV inhibitor, as previously reported [33], [34].

### Intracellular GLP-1 and glucagon content quantification

After the GLP-1 and the glucagon secretion experiments, the cells or rat pancreatic islets were incubated in a solution of HCl (10 N) and ethanol (70%) and shaken overnight at 4°C. The samples were then collected in vials and kept frozen at −20°C until the analysis. Insoluble material was removed by centrifugation. For the determination of the GLP-1 content [total GLP-1 (7–36 and 9–39)] we used a specific ELISA kit (Millipore Corp. Billerica, MA, U.S.A.), that detects GLP-1 (7–36) and (9–36) and has no significant cross-reactivity with GLP-2, GIP, Glucagon and Oxyntomodulin. For the determination of the Glucagon content we used a specific RIA Kit (Millipore Corp. Billerica, MA, U.S.A.) that detects glucagon and has no significant cross-reactivity with Oxyntomodulin [31].

### Preparation of cell lysates for protein expression analyses

The samples for protein expression analyses were prepared as previously described [35], [36]. Briefly, at the end of the culture period, the cells were washed twice in Phosphate Buffered Saline (PBS) and lysed in ice-cold modified Radio-ImmunoPrecipitation Assay (RIPA) buffer. The adherent cells were scraped off the dish and the suspension was transferred into a centrifuge tube. The samples were then homogenized by sonication in RIPA buffer. After sonication, the lysate was centrifuged at 10,000× *g* in a pre-cooled centrifuge (4°C) for five minutes. The supernatant was immediately transferred to a fresh centrifuge tube and the pellet was discarded. The protein concentration was determined by BCA assay (Thermo Scientific, Pierce, Meridian Rd, Rockford, IL U.S.A.) according to the manufacturer's instructions.

### Western blot analysis

Aliquots of cell lysates were subjected to Western blot analysis. Western blot analyses of crude lysates were performed as described previously [37]. Briefly, after protein normalization (as determined by the BCA assay), the proteins were diluted and subjected to SDS-PAGE. The resolved proteins were then transferred to nitrocellulose membranes, immunoblotted with specific antibodies and revealed with an enhanced chemiluminescence method. The nitrocellulose membrane was then stripped with Restore stripping buffer (Thermo Scientific, Pierce, Meridian Rd, Rockford, IL, U.S.A.) for 12 minutes at room temperature and subsequently re-probed with the anti-actin monoclonal antibody. All immunoblot signals were visualized with the ECL method (Amersham, Little Chalfont, U.K.), auto-radiographed and subjected to densitometric analysis.

### Densitometric analysis

The densitometric analysis was performed using *ImageJ* ™ software version 1.41 was used (free download available at http://rsbweb.nih.gov/ij/download.html). The subsequent data analyses were performed with GraphPad Prism version 5.0 (GraphPad Software Inc., La Jolla, CA, U.S.A.).

### mRNA isolation and cDNA synthesis

Total RNA was extracted with TRIzol reagent (Invitrogen, Carlsbad, CA, U.S.A) according to the manufacturer's instructions, purified with ribonuclease-free deoxyribonuclease I (Sigma-Aldrich, St. Louis, MO, U.S.A.) and quantified by spectrophotometry. First-strand cDNA was produced from total RNA by using ThermoScript RT and random primers (Invitrogen, Life Technologies, Monza, Italy) according to the manufacturer's instructions. Briefly, 2 µg of total RNA (400 ng/µL) was diluted in a mix containing 1 µL random primer (50 ng/µL), 7 µL of cDNA Synthesis Mix (5X First-Strand Buffer 4 µL, dNTP Mix (10 mM each) 1 µL, DTT 0.1 M 1 µL, RNaseOUT 1 µL, ThermoScript RT 1 µL) and the volume was adjusted to 20 µL with diethylpyrocarbonate (DEPC)-treated water. The 0.2 mL tubes were incubated at 30°C for 10 min, 42°C for 60 min, the reaction was terminated by heating at 99°C for 5 min and cooling it at 5°C for 5 min.

For the full-length *glucagon-like peptide 1 receptor (Glp1r)* analysis, RNA samples were converted to complementary DNA (cDNA) full lengths by reverse transcription using SuperScript III and Oligo dT reagents (Invitrogen) according to the manufacturer's instructions. The SuperScript III enzyme is usually used to synthesize cDNA at a temperature range of 42–55°C, providing increased specificity, higher yields of cDNA and more full-length product than other reverse transcriptases, as reported in the datasheet.

### RT-PCR amplification and Real-time quantitative PCR

Full lengths mouse and hamster *glucagon-like peptide 1 receptor (Glp1r)* transcripts were obtained by polymerase chain reaction (PCR) with primer 5′-ATGGCCAGCACCCCAAGCCTCC-3′ and 5′-TCAGCTGTAGGAACTCTGG-3′ relative to the mouse *Glp1r* sequence (NM_021332.2), Gene ID: 14652; 5′-ATGGTTTCCTTCACGTCGG-3′ and 5′-TCAGCTGCAGGAACCCTGG-3′ relative to the hamster *Glp1r* sequence (XM_003504450), Gene ID: 100763400.

Forward 5′-CCACCATGTACCCAGGCATT-3′ and reverse 5′-AGGGTGTAAAACGCAGCTCA-3′ primers specific to mouse *actin beta (Actb)* sequence (NM_007393.3), Gene ID: 11461, were used in control analyses as housekeeping gene.

Forward 5′-GCTCTTTTCCAGCCTTCCTT-3′ and reverse 5′-GAGCCAGAGCAGTGATCTCC-3′ primers, specific to hamster *Actb* sequences (NM_001244575.1), Gene ID: 100689477, were used as the internal control of gene expression. With these primer pairs the expected bands for *Glp1r* were respectively 1392 bp for mouse (α-TC1-6) and 1602 bp for hamster (InR1G9); the expected bands for *Actb* were respectively 253 bp for mouse (α-TC1-6) and 187 bp for hamster (InR1G9). Aliquots of each amplified were analyzed by electrophoresis on 1.5/2% agarose gels and visualized by SYBR Safe DNA gel stain (Invitrogen).

Quantitative Real-time PCR was performed using SYBR Green PCR Master Mix reagents (Applied Biosystems, Branchburg, NJ, U.S.A.) in an ABI PRIMS 7700 (PE Applied Biosystems, Foster City, CA). The reaction mixture was obtained by mixing 3 µL of cDNA [pre-diluted 1∶8 with DEPC-treated water and corresponding to 37.5 ng of RNA], diluted in 8.5 µL of DEPC-treated water, 12.5 µl Power SYBR Green PCR Master Mix (2×) (Applied Biosystems, Branchburg, NJ, U.S.A.), and 0.5 µL of gene-specific primers (10 µM) in a final reaction volume of 25 µL. The cycling conditions were as follow: a denaturation step at 95°C for 10 minutes, followed by 40 cycles of 95°C for 15 seconds, 60°C for 1 minutes and a final step for the generation of a dissociation curve to distinguish between the main PCR product and primer-dimer.

Primer Express software (Applied Biosystems) was used to design appropriate primer pairs, which were then synthesized by MWG-Biotech (Ebersberg, Germany). Gene amplification was performed using the following primers: 5′-TTGCCTTTGTGATGGACGAA-3′ and 5′-AGGAAGTGAAGGAGAGTTCTGTGAA-3′ specific for mouse *glucagon-like peptide 1 receptor* (*Glp1r*; NM_021332.2), Gene ID: 14652; 5′-CCTGGTTGGTATCCCGGGA-3′ and 5′- CCGCTTCAGCTGAAGTCGCA-3′ specific for mouse *Pax6* (XM_006498913.1), Gene ID: 18508; 5′- CCCTTCAAGACACAGAGGAGAA-3′ and 5′-TCTCGCCTTCCTCGGCCTTTCA-3′ specific for mouse *glucagon gene* (*Gcg*; NM_008100.3), Gene ID: 14526; 5′-GCCAACCGTGAAAAGATGACC-3′ and 5′-TGCCGATAGTGATGACCTGACC-3′ specific for mouse *actin beta* (*Actb*; NM_007393.3), Gene ID: 11461, as housekeeping gene. Each sample was run in triplicate. When the difference between the triplicates was greater than 0.5 Ct, the assay was repeated.

β-actin was used as an endogenous control for normalization. The expression was almost equally expressed in all the different experimental settings. The mean of Ct values for β-actin was 14.7±0.3 in all samples analyzed, while the mean of Ct values for GLP-1 receptor was 20.6±0.4 in β-TC1 cells and 26.6±0.3 in α-TC1-6 cells.

The gene expression changes were determined with the Comparative CT (2^−ΔΔCt^) method (Applied Biosystems) for Real-Time PCR.

### Immunofluorescence studies

Twenty-four hours after planting, the α-TC1-6 cells were fixed in PBS containing 4% paraformaldehyde for 15 min at room temperature, and then incubated with a blocking solution (normal goat serum 5%, Triton 0.3% in PBS) for 1 h. Fixed materials were incubated overnight at 4°C with the anti-GLP-1R antibody in PBS supplemented with 1% BSA (wt/vol) and 0.3% Triton X-100. After rinsing, cells were incubated in Alexa Fluor-549 anti-Rabbit IgG secondary antibody (Invitrogen, Monza MB, Italy) for 1 h at room temperature. Cells were finally counterstained with Hoechst 33258 to color the nuclei and phalloidin to stain the cytoskeleton. Images were digitally acquired with epifluorescence microscopy using an Orca charge-coupled device Camera (Hamamatsu City, Shizuoka, Japan) and processed with Image-Pro Plus 4.0 software (Media Cybernetics, Silver Spring, MD) [38].

### Statistics

The differences between means of unpaired samples were analyzed with Student's *t* test. Comparisons between multiple means were evaluated by ANOVA test followed by *post hoc* analysis of significance (Bonferroni test). For both tests the level of significance was set at *p*<0.05.

Statistical analysis was performed with GraphPad Prism 5.0 (GraphPad Software, Inc., San Diego, CA). Data are expressed as mean ± SEM (SE).

## Results

### Full length Glp1r transcript expression

In order to evaluate the presence of *Glp1r* in α-TC1-6 cells, we performed RT-PCR amplification using specific primers for *Glp1r* gene.

**Figure 1:**
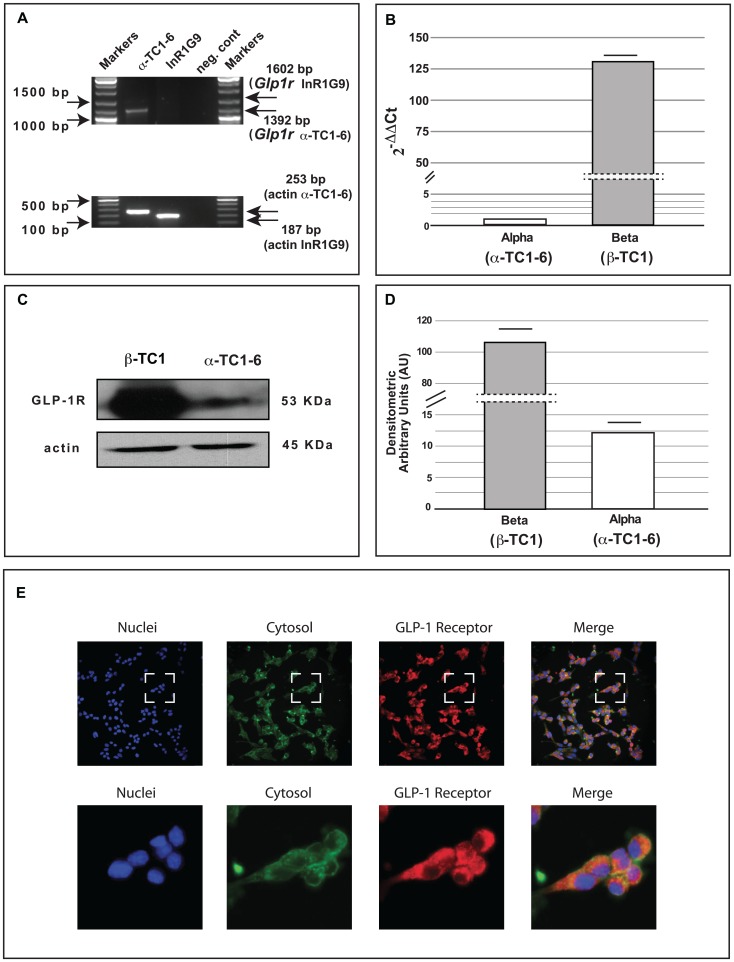
GLP-1 receptor expression in alpha cells (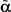
TC1-6 and InR1G9) and in beta cells (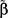
TC1). (A) Full length *Glp1r* transcript expression as analysed by RT-PCR in α-TC1-6 and in InR1G9 cells. (B) *Glp1r* gene expression as analysed by Real Time PCR (means of five experiments) in beta cells (β-TC1 cells as positive control) and alpha cells (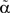
TC1-6); data are expressed as (2^−ΔΔCt^) considering as 1 the *Glp1r* gene expression in alpha cells (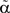
TC1-6) and using mouse *actin beta (Actb)* as housekeeping gene. (C) Representative Western Blot of GLP-1R protein expression in β-TC1 cells and in 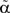
TC1-6 cells. (D) Densitometric analysis of GLP-1R protein expression in β-TC1 cells and in 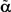
TC1-6 cells. The data are the means of five different experiments ± SE. (E) GLP-1R (*red*) immunoreactivity in 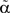
TC1-6 cells. Hoechst 33258 was used to visualize nuclei (*blue*) and phalloidin to stain the cytoskeloton (*green*). Squares (upper side) indicate the areas shown at higher magnification (lower side).

The full-length *Glp1r* transcript expression was analysed by RT-PCR amplification in α-TC1-6 cells and in InR1G9 cells, a hamster-derived alpha cell line that lack GLP-1R. We found that *Glp1r* was expressed in α-TC1-6 cells, while it was not detectable in InR1G9 cells (Figure 1, Panel A).

### GLP-1 receptor, gene and protein expression in α-TC1-6 cells

To further investigate the expression of GLP-1R in α-TC1-6 cells, we performed Real-Time PCR and Western Blot analyses. We detected both GLP-1R mRNA and protein, although at levels far lower than those found in βTC1 cells (Figure 1, Panel B, C, D).

To address whether in our model all the cells or only a subset of them express the GLP-1R, immunofluorescence staining for the GLP-1R was performed (Figure 1, Panel E). Positive immunoreactivity for the GLP-1R was observed on almost all cells.

### The effect of GLP-1 on cAMP levels

**Figure 2:**
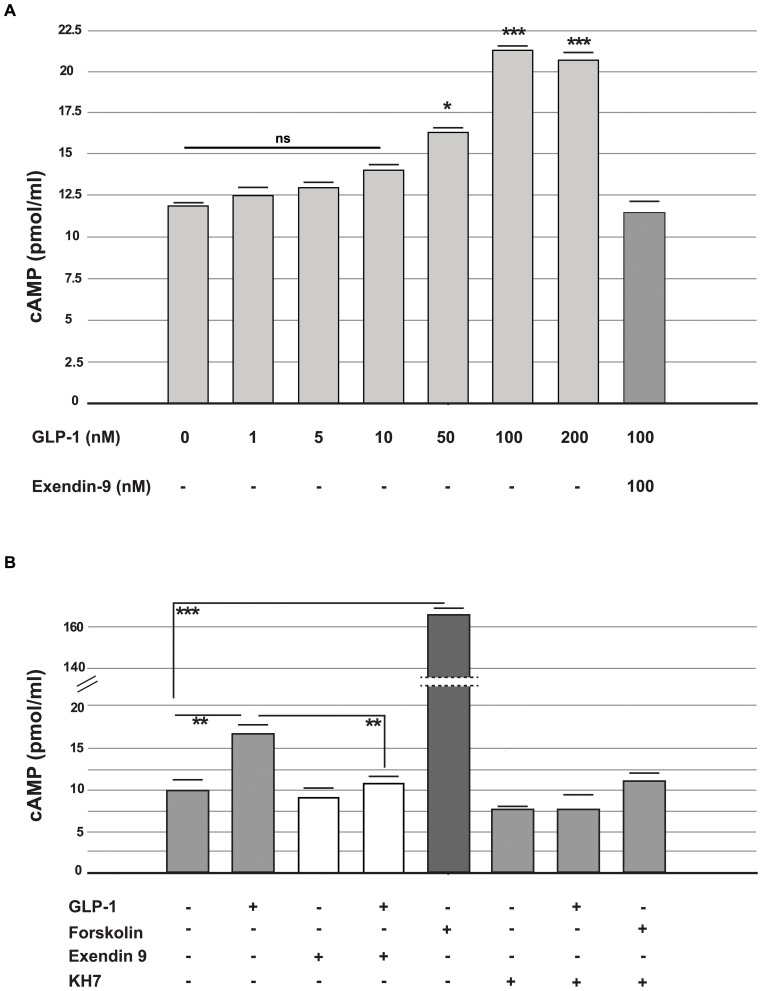
cAMP levels in alpha cells (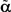
TC1-6). cAMP intracellular levels as measured by ELISA analysis. Upper side (panel A) shows a dose-response to GLP-1 acute stimulation (2 hours) at 25 mmol/l glucose concentration. Lower side (panel B) shows cAMP levels in cells chronically cultured (72 hours) in the presence or absence of GLP-1 and during the last 12 hours with or without GLP-1 (100 nmol/l), Exendin-9 (100 nmol/l), Forskolin (50 nmol/l) and KH7 (25 µmol/l) alone or in combination. The cells were then washed twice and incubated for two hours in Krebs-Ringer buffer (KRB) containing 25 mmol/l of glucose and 0.5% BSA (pH = 7.4) in the presence or absence of GLP-1 (100 nmol/l), Exendin-9 (100 nmol/l), KH7 (25 µmol/l) Forskolin (50 nmol/l) or KH7 (25 µmol/l) alone or in combination. The data are the means of five different experiments. ns (p>0.05): not statistically significant; * p<0.05; ** p<0.01; ***p<0.001, using one-way ANOVA followed by Bonferroni test.

To assess the biological activity of GLP-1 in α-TC1-6 cells, we first measured intracellular cAMP levels. To determine the acute effect of GLP-1, cells were grown in the absence of GLP-1 for 72 hours then washed and subsequently exposed to GLP-1 (from 0 to 200 nmol/l) alone or in co-presence of Exendin-9 for two hours; subsequently, the intracellular cAMP levels were measured. Acute GLP-1 exposure increased intracellular cAMP levels with maximum effect at 100 nmol/l dose (21.94±2.1 *vs.* 12.14±1.1 pmol/ml in control cells; n = 5; p<0.001 *vs.* control) (Figure 2 A).

Next, the cAMP levels were measured in cells that had been exposed to GLP-1 (100 nmol/l) for 72 hours. The cAMP levels were significantly higher in the cells that had been exposed to GLP-1 than in the control cells (p<0.01; n = 5) (Figure 2 B). Additionally, we measured the cAMP levels in cells cultured in the presence of both GLP-1 and Exendin-9 (100 nmol/l), a competitive antagonist of the GLP-1 receptor. Under these experimental conditions, Exendin-9 inhibited the stimulatory effect of GLP-1 (Figure 2 B). We also investigated the effects of Forskolin (50 nmol/l), a direct cAMP inducer, and KH7 (25 µmol/l), a selective inhibitor of soluble adenylyl cyclase. As expected, Forskolin significantly increased intracellular cAMP levels. In contrast, KH7 alone had little or no effect on cAMP levels. However, when co-incubated with either GLP-1 or Forskolin, KH7 significantly reduced the effects of both of these proteins on cAMP levels (Figure 2 B).

### The effects of GLP-1 on glucagon secretion and content in αTC1-6 cells

After acute GLP-1 exposure (100 nmol/l for two hours) glucagon release was found to be lower in cells exposed to GLP-1 compared to control cells (256 fmol/µg protein/hour ±5 *vs.* 358 fmol/µg protein/hour ±8 in control cells; n = 5; p<0.001).

**Figure 3:**
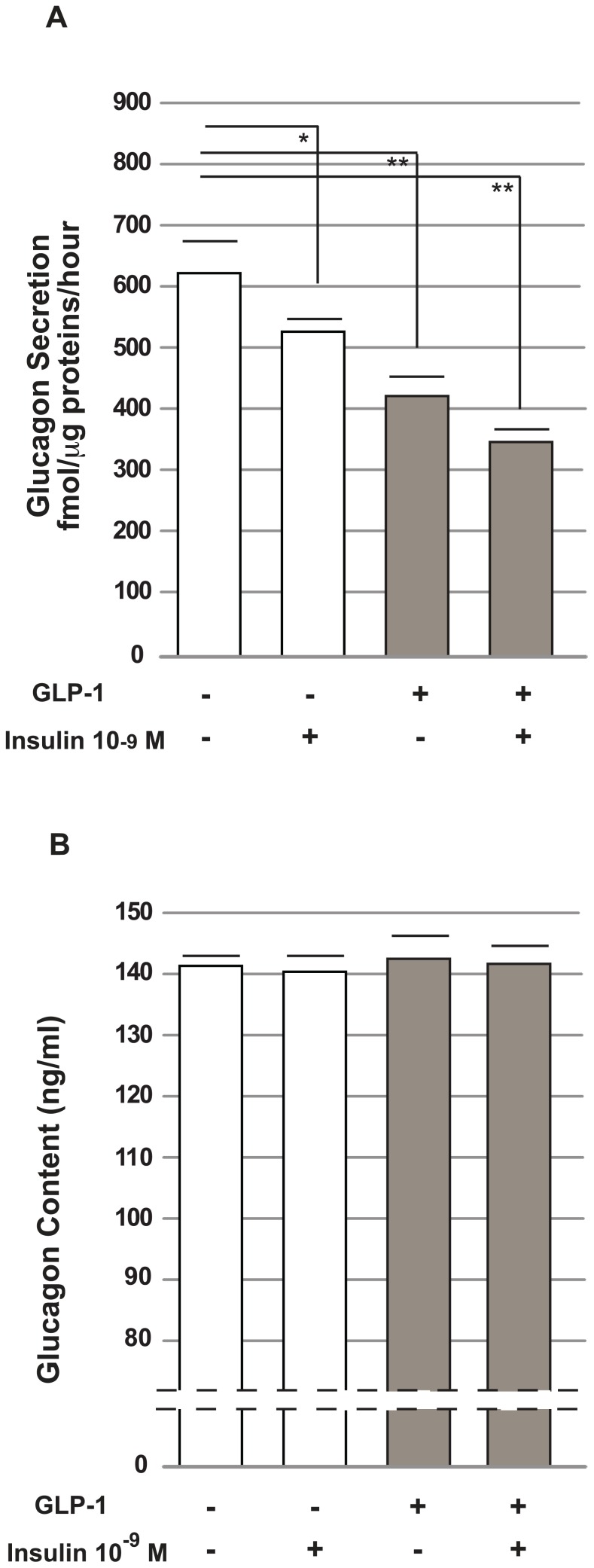
Glucagon secretion and glucagon content in alpha cells (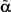
TC1-6). (A) Glucagon secretion in α-TC1 cells. The glucagon levels were measured in cells cultured with or without GLP-1 for 72 hours, washed and cultured for two more hours in KRB (glucose 25 mmol/l) in the presence or absence of GLP-1 (100 nmol/l) and/or insulin (10^−9^ M) for five minutes. The data are expressed as fmol/µg protein/hour; the means ± standard error (SE), n = 5; * p<0.05; ** p<0.01; ***p<0.001, using one-way ANOVA followed by Bonferroni test. (B) The intracellular glucagon content in α-TC1 cells. The cells were cultured in DMEM, with or without GLP-1 (100 nmol/l) for 72 hours, washed and cultured for two hours in KRB (glucose 25 mmol/l) in the presence or absence of GLP-1 (100 nmol/l) and/or insulin (10^−9^ M) for the last five minutes. The cells were then lysed in 0.1 M HCl and assayed for glucagon content. The data are expressed as ng/ml; means ± SE, n = 5.

To investigate the effects of chronic exposure to GLP-1 (100 nmol/l) on glucagon secretion, we measured glucagon secretion in control cells and in cells grown for 72 hours in the presence of GLP-1. After chronic exposure to GLP-1, the cells were washed and cultured for two more hours in the presence or absence of GLP-1 (100 nmol/l). Glucagon released was then measured. As shown in Figure 3A, glucagon secretion was significantly lower in cells that were exposed to GLP-1 for a prolonged period of time than in control cells (p<0.01). This effect was stronger than the effect observed when cells were exposed to insulin (10^−9^ M, for five minutes), a physiological modulator of glucagon secretion (p<0.05, insulin groups *vs.* control groups). The combination of GLP-1 and insulin produced no additive effect. In contrast to glucagon secretion, the glucagon content was similar in the cells cultured in the presence or absence of GLP-1 or insulin (Figure 3B).

### The effects of GLP-1 on the MAPKs pathway

**Figure 4:**
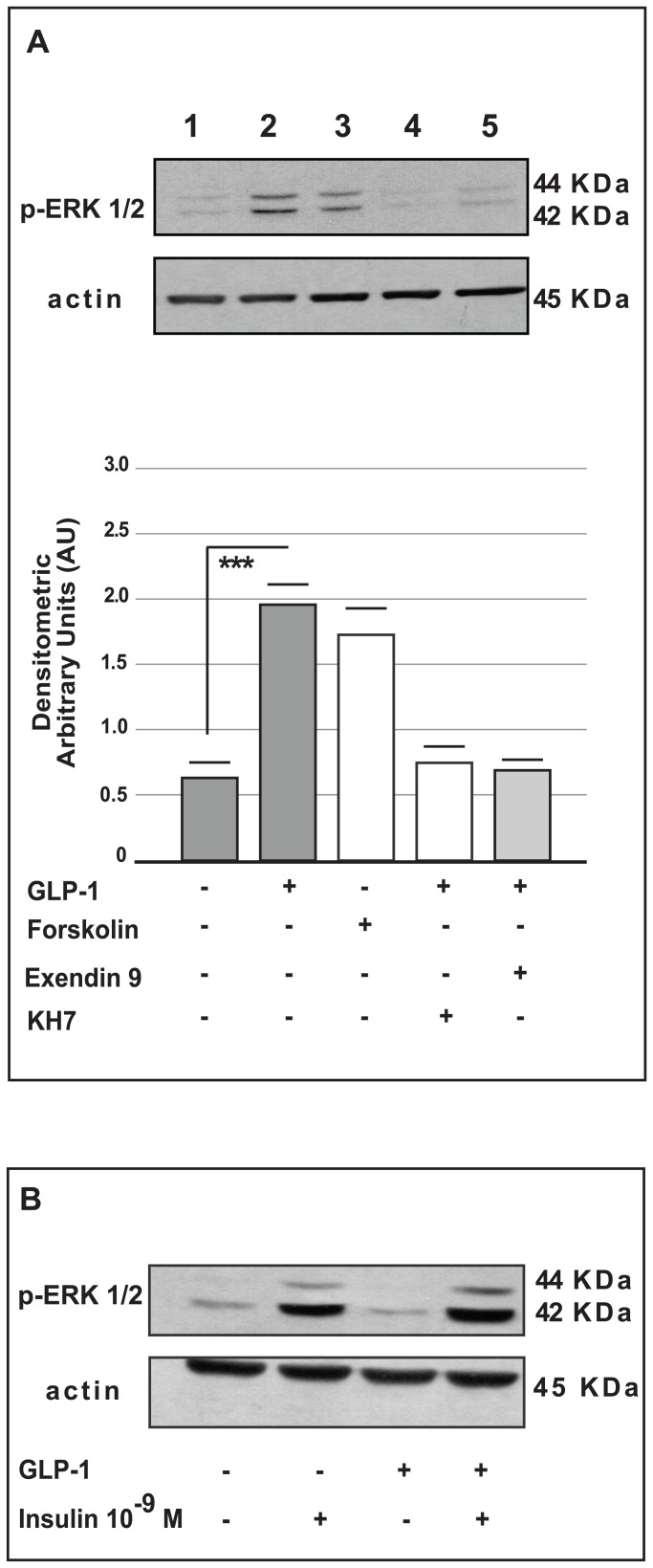
Western blot analysis for Erk 44/42^MAPK^ phosphorylation in alpha cells (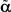
TC1-6 and InR1G9). The upper side of panel (A) shows a representative Western blot for Phospho-p44/42 MAPK (Erk1/2) (Thr202/Tyr204) and for actin in 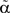
TC1-6 cells: control cells (line 1), in cells cultured in the presence of GLP-1 (100 nmol/l) for 72 hours (line 2), in cells treated with adenylyl cyclase activator (Forskolin 50 nmol/l) for 72 hours (line 3), in cells treated with GLP-1 (100 nmol/l) and an adenylyl cyclase inhibitor (KH7 25 µmol/l) for 72 hours (line 4) and in cells cultured for 72 hours in the co-presence of GLP-1 (100 nmol/l) and Exendin-9 (100 nmol/l) (line 5). The lower side shows the densitometric analysis from five different experiments. The data are expressed as the means ± SE. *** p<0.001 *vs*. control groups, using one-way ANOVA followed by Bonferroni test. (B) Western blot analysis for Phospho-p44/42 MAPK (Erk1/2) (Thr202/Tyr204) and actin in InR1G9 cells cultured in the presence or absence of GLP-1 (100 nmol/l) for 72 hours, washed and acutely stimulated with insulin (10^−9^ M) for the last five minutes in the presence or absence of GLP-1 (100 nmol/l).

Because cAMP is known to regulate the MAPKs pathway [29], [39], we investigated the ability of GLP-1 to activate this intracellular pathway. Phospho-p44/42^MAPK^ (ERK 1/2) was significantly higher in α-TC1-6 cells chronically cultured with GLP-1 than in control cells (p<0.001 *vs.* control, Figure 4A). To further investigate the direct interaction between GLP-1-mediated cAMP activation and p44/42^MAPK^ phosphorylation, we performed experiments using Forskolin (50 nmol/l), an adenylyl cyclase activator, and KH7 (25 µmol/l), an adenylyl cyclase inhibitor. The phosphorylation of p44/42^MAPK^ was significantly increased in α-TC1-6 cells cultured in the presence of Forskolin (Figure 4A). In contrast, p44/42^MAPK^ protein phosphorylation was lower in cells cultured in the co-presence of GLP-1 and KH7 (25 µmol/l) than in cells exposed to GLP-1 alone (Figure 4A).

Next, we investigated whether the effect of GLP-1 was mediated by the GLP-1 receptor. We performed experiments in the presence of Exendin-9 (100 nmol/l), a competitive antagonist of the GLP-1 receptor. The phosphorylation of p44/42^MAPK^ was unaffected in the α-TC1-6 cells co-cultured for 72 hours with GLP-1 and Exendin-9, and the phosphorylation levels in these cells were similar to those of the control cells (Figure 4A).

Finally, we performed experiments in another pancreatic alpha cell line (InR1G9) that lacks GLP-1 receptor and related biological activity [20]–[23]. In the InR1G9 cell line, GLP-1 exposure (100 nmol/l up to 72 hours) did not alter p44/42 ^MAPK^ phosphorylation, as determined by Western blot analysis (Figure 4 B). In contrast, exposure to insulin (10^−9^ M) for five minutes activated p44/42^MAPK^ in these cells.

### The effect of GLP-1 on Pax6 gene and protein expression

**Figure 5:**
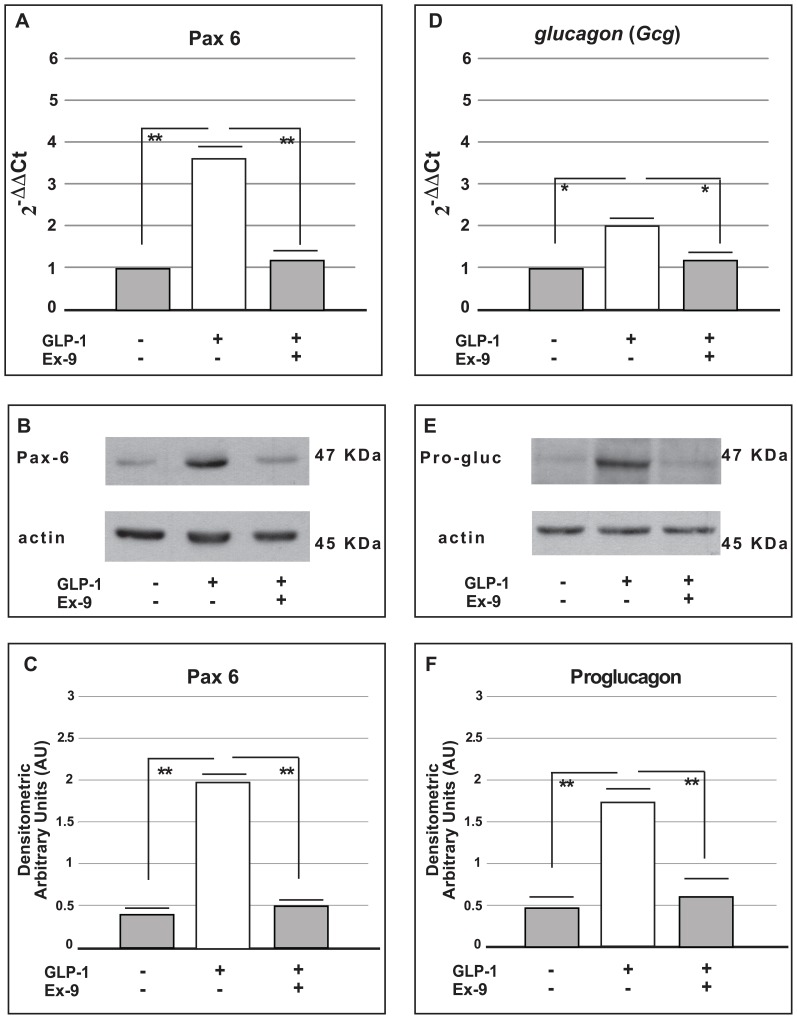
Real-time PCR analysis and Western blot analysis for *Pax6* and *glucagon (Gcg)* gene and protein expression in alpha cells (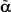
TC1-6). Panel (A) shows the expression of *Pax 6* as determined by Real-Time PCR analysis (means from five different experiments) in control cells and in cells cultured for 72 hours in the presence of GLP-1 (100 nmol/l) alone or in combination with Exendin-9 (100 nmol/l). Panel (B) shows a representative Western Blot for the Pax6 protein expression in control cells and in cells cultured for 72 hours in the presence of GLP-1 (100 nmol/l) alone or in combination with Exendin-9 (100 nmol/l); Panel (C) shows the densitometric analysis (means from five different Western Blot experiments). The data are expressed as means ± SE. ** p<0.01, using one-way ANOVA followed by Bonferroni test. Panel (D) shows the expression of the *glucagon* (*Gcg*) gene as determined by Real-Time PCR analysis (means from five different experiments) in control cells and in cells cultured for 72 hours in the presence of GLP-1 (100 nmol/l) alone or in combination with Exendin-9 (100 nmol/l). Panel (E) shows a representative Western Blot for proglucagon protein expression in control cells and in cells cultured for 72 hours in the presence of GLP-1 (100 nmol/l) alone or in combination with Exendin-9 (100 nmol/l). Panel (F) shows the densitometric analysis (means from five different Western Blot experiments). The data are expressed as the means ± SE. * p<0.05; ** p<0.01, using one-way ANOVA followed by Bonferroni test.

Because the MAPK signalling pathway regulates Pax6 expression through P38^MAPK^ activation [19], [40], we next investigated the effects of GLP-1 on *Pax6* expression. *Pax6* is a transcription factor that is required for the normal development of several organs, including the pancreas and pancreatic alpha cells. Additionally, it is involved in the expression of *Gcg* genes [41]–[43]. *Pax6* gene and protein expression was analysed by Real-Time PCR and Western blot, respectively. In our experimental model, *Pax6* gene expression was significantly increased in cells cultured in the presence of GLP-1 (100 nmol/l) for 72 hours (Figure 5, panel A). In these cells, Pax6 protein expression was also higher than in the control cells (p<0.01 *vs.* control groups) (Figure 5, panel B and C).

To further investigate the ability of GLP-1 to induce Pax 6 gene and protein expression in alpha cells, we also cultured α-TC1-6 cells in the co-presence of GLP-1 and Exendin-9 (a specific GLP-1R antagonist). As expected, Exendin-9 inhibited the stimulatory effect of GLP-1 on Pax 6 expression (Figure 5).

**Figure 6:**
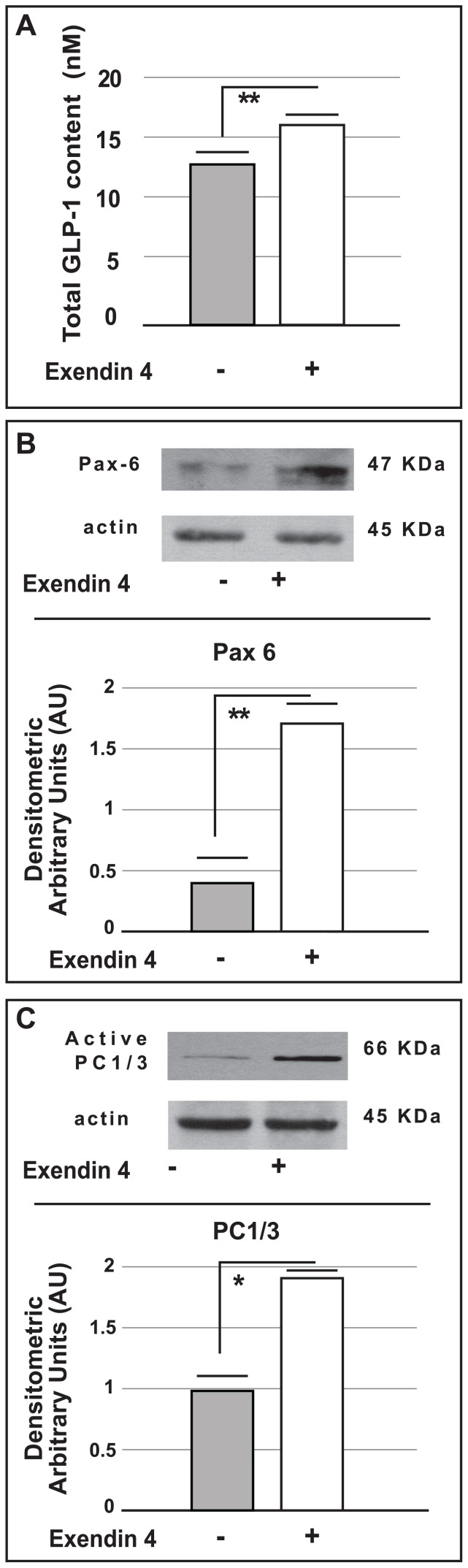
GLP-1 total content and Western Blot analysis for Pax6 and PC1/3 in pancreatic rat islets chronically exposed to Exendin-4. Panel (A) shows the total intracellular GLP-1 content of pancreatic rat islets cultured in the presence or absence of Exendin-4 (100 nmol/l) for 72 hours. The data are expressed as means ± SE. ** p<0.01 *vs.* control group. Panel (B) the upper side shows a representative Western Blot for the Pax6 protein in rat pancreatic islets cultured in the presence or absence of Exendin-4 (100 nmol/l) for 72 hours; the lower side shows the densitometric analysis (means from five different Western Blot experiments). The data are expressed as means ± SE. ** p<0.01 *vs*. control groups. Panel (C) the upper side of shows a representative Western Blot for PC1/3 protein expression in pancreatic rat islets cultured in the presence or absence of Exendin-4 (100 nmol/l) for 72 hours; the lower side shows the densitometric analysis (means from five different Western Blot experiments). The data are expressed as the means ± SE. * p<0.05 *vs.* control groups.

We also measured Pax6 protein expression in rat pancreatic islets cultured for 72 hours in the presence or absence of Exendin-4 (100 nmol/l). Under these conditions Pax6 expression was found to be significantly higher in islets chronically cultured with Exendin-4 (p<0.01 *vs.* control groups) (Figure 6).

### The effect of GLP-1 on glucagon (Gcg) gene and protein expression

Because Pax6 controls glucagon (*Gcg*) gene expression [19], we measured the expression of the *Gcg* gene and proglucagon protein in cells treated with GLP-1. The expression of the *Gcg* gene and proglucagon protein was significantly increased in cells cultured for 72 hours with GLP-1 (100 nmol/l) (Figure 5, panels D, E and F). To further investigate the capability of GLP-1 to induce Pax 6 and then proglucagon expression, we measured *Gcg* gene and proglucagon protein expression also in α-TC1-6 cells cultured in the co-presence of GLP-1 and Exendin-9 (a specific GLP-1R antagonist). As expected, Exendin-9 markedly reduced GLP-1-induced proglucagon protein expression (Figure 5).

### The effect of GLP-1 on the expression of the pro-hormone convertases PC1/3 and PC2 after GLP-1 stimulation

**Figure 7:**
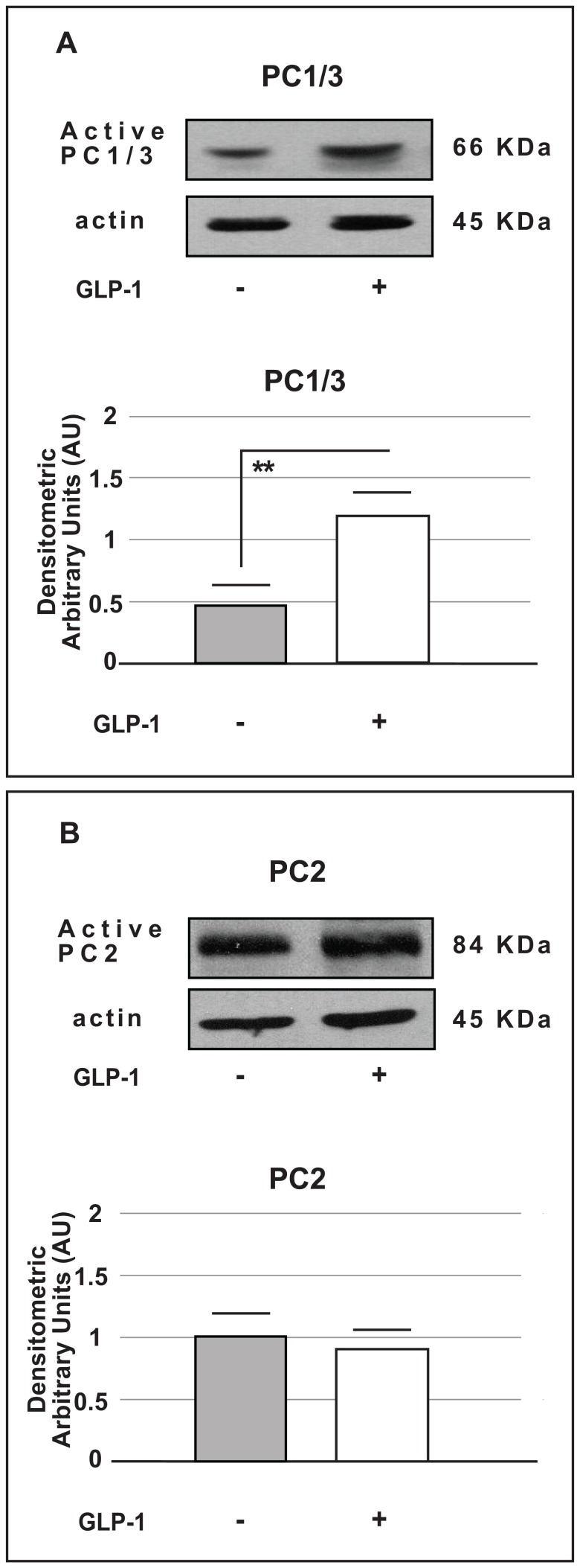
Western blot analysis for PC1/3 and PC2 protein expression in alpha cells (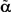
TC1-6). Western blot analysis of active PC1/3 (A) and PC2 (B) protein expression in control cells and in cells cultured for 72 hours in the presence of GLP-1 (100 nmol/l). The upper sides of the panels show representative Western Blots for active PC1/3 or active PC2. The lower sides of the panels show the densitometric analysis (means from five different Western Blot experiments). The data are expressed as the means ± SE. ** p<0.01 *vs*. control group.

Because the expression of the *Gcg* gene and proglucagon protein was increased but the glucagon content was unchanged in α-TC1-6 cells cultured with GLP-1, we hypothesized that regulators of glucagon (such as converting enzymes) may be affected by GLP-1. It is known that PC2 (pro-enzyme convertase 2) activity produces glucagon in alpha cells and PC1/3 (pro-enzyme convertase 1/3) activity generates GLP-1 in intestinal L cells [44], [45]. Recent evidence indicates that PC1/3 is also expressed in human pancreatic alpha cells and that GLP-1 was detectable in human islets [15]. On the basis of these findings, we measured the protein expression of PC1/3 and PC2 in our system. Under our experimental conditions, PC1/3 protein expression was markedly higher in cells exposed to GLP-1 than in control cells (Figure 7, panel A). In contrast, PC2 protein expression was not affected by chronic GLP-1 exposure (Figure 7, panel B).

In addition we analysed PC1/3 protein expression also in rat pancreatic islets cultured for 72 hours with Exendin-4 (100 nmol/l); similarly to our alpha cell model, PC1/3 protein content was significantly increased in islets chronically exposed to Exendin-4 (p<0.05 *vs.* control), (Figure 6).

### GLP-1 content and secretion were enhanced in α-TC1-6 cells and in islets that were chronically exposed to GLP-1

Next, we measured GLP-1 content and secretion after exposure to GLP-1 (100 nmol/l) for 72 hours.

**Figure 8:**
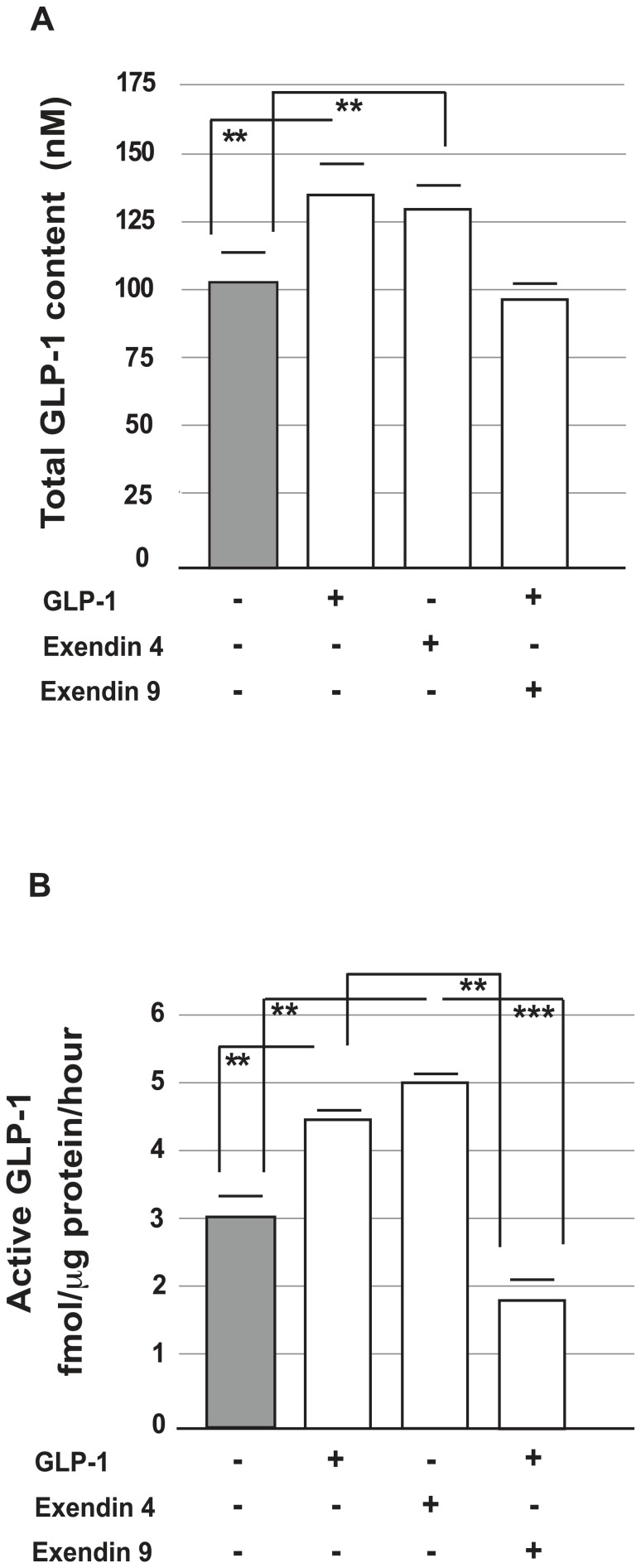
GLP-1 (active and total) quantification in cells (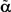
TC1-6) chronically exposed to GLP-1, Exendin-4 or Exendin-9. Panel (A) shows the total intracellular GLP-1 content of α-TC1-6 cells cultured in the presence or absence of GLP-1 (100 nmol/l), Exendin-4 (100 nmol/l), Exendin-9 (100 nmol/l) or Exendin-9 (100 nmol/l) in co-presence of GLP-1 (100 nmol/l) for 72 hours. The data are expressed as means ± SE. ** p<0.01, using one-way ANOVA followed by Bonferroni test. Panel (B) shows the acute secretion of active GLP-1 by α-TC1-6 cells cultured in the presence or absence of GLP-1 (100 nmol/l), Exendin-4 (100 nmol/l), Exendin-9 (100 nmol/l) or Exendin-9 (100 nmol/l) in co-presence of GLP-1 (100 nmol/l) for 72 hours. After culture, the cells were washed in GLP-1-free Krebs-Ringer buffer and incubated for two more hours in Krebs-Ringer buffer containing 16.7 mmol/l of glucose and 0.5% BSA (pH 7.4) in the absence of GLP-1 (100 nmol/l) Exendin-4 (100 nmol/l) or Exendin-9 (100 nmol/l). The data are expressed as the means ± SE. ** p<0.01, *** p<0.001 using one-way ANOVA followed by Bonferroni test.

Under these conditions, the presence of intracellular GLP-1 was found to be significantly higher in cells cultured with GLP-1 than in control cells (Figure 8 A).

We also investigated the acute secretion of GLP-1. After 72 hours of exposure to GLP-1, the cells were washed twice in GLP-1-free Krebs-Ringer buffer (KRB) and incubated for two more hours in Krebs-Ringer buffer containing 16.7 mmol/l glucose and 0.5% BSA (pH 7.4) in the absence of GLP-1.

Surprisingly, the α-TC1-6 cells that had been cultured with GLP-1 released significantly more of the active form of GLP-1 than the control cells (p<0.01 *vs.* control) (Figure 8 B).

To further investigate the capability of GLP-1 to induce GLP-1 synthesis and release (positive feed-back)

in alpha cells, we cultured α-TC1-6 cells also in the presence of Exendin-4 (a specific GLP-1 analogue that acts as agonist of GLP-1R), or in the co-presence of GLP-1 and Exendin-9 (a specific GLP-1R antagonist).

We observed that Exendin-4 stimulatory effect on GLP-1 secretion and content in α-TC1-6 was similar to that of GLP-1 and that Exendin-9 inhibited the stimulatory effect of GLP-1 (Figure 8).

We also measured GLP-1 secretion and content in isolated rat islets cultured for 72 hours in the presence or absence of Exendin-4 (100 nmol/l).

Under these conditions, the presence of intracellular GLP-1 was significantly higher in islets chronically cultured with Exendin-4 (p<0.01 *vs.* control cells) (Figure 6).

Although at a very low levels, also in rat pancreatic islets we could observe an acute secretion of active GLP-1 (data not shown).

## Discussion

Our data provide evidence of a chronic and direct effect of GLP-1 on the α-TC1 clone 6 pancreatic cell line. These cells have been previously used to study glucagon secretion and gene expression [17]-[19] and, in respect to primary islets, represent a homogeneous cellular population. We detected GLP-1 receptor (GLP-1R) in this cell line, although at considerably lower levels than in pancreatic beta cells, as previously reported by others [14]. Although at the present time there is currently no known specific GLP-1R antibody that detects authentic GLP-1R protein [46], [47], we clearly found not only GLP-1R (protein and mRNA transcript) but also distinct biologic effects, such as the increase in cAMP levels and the inhibition of the release of glucagon. Moreover, in our model, the GLP-1 receptor antagonist Exendin-9 prevented these effects. The heterogeneity in the current data in the literature could be because not all pancreatic alpha cell lines, and not all alpha cells in islets, express the GLP-1 receptor.

These initial experiments convinced us that this cell line was a suitable model for understanding how alpha cells react to prolonged exposure to high GLP-1 levels (as experienced by type 2 diabetes patients treated with DPP-IV inhibitors or GLP-1 analogues). These questions are critical to the field of diabetes therapy, as there has been much controversy in the recent literature surrounding the benefits and risks of incretin-based therapy in humans [48]. We incubated α-TC1-6 cells in the presence of GLP-1 and measured the intracellular cAMP levels and the ability of GLP-1 to activate the MAPKs pathway. We found that alpha cells exposed to GLP-1 for a prolonged period had higher cAMP levels and higher levels of phospho-Erk 44/42 than control cells, indicating that the MAPK pathway had been activated in cells exposed to GLP-1. This effect, which was inhibited by Exendin-9, was presumably due to the binding of GLP-1 to the GLP-1 receptor and mediated by cAMP (as indicated by the inhibition of the effect by KH7). Since it is known that glucagon secretion in isolated alpha cells occurs at high glucose levels [49], [50] we also performed some experiments (glucagon secretion and cAMP production) at high glucose levels (25 mmol/L). Under these condition GLP-1 was able to inhibit glucagon secretion better than insulin and induce cAMP production.

Because the MAPK signalling pathway has been reported to regulate Pax6, a transcription factor known to control *Gcg* gene and proglucagon synthesis, we measured the expression of Pax6 and proglucagon [19], [40]. We found that both the gene and protein expression of Pax6 and proglucagon was increased in cells exposed to GLP-1 for a prolonged period. However, even though the levels of *Gcg* gene and proglucagon protein were increased, the glucagon content of these cells was unaffected. To better understand this finding, we hypothesized that because GLP-1 is a product of the *Gcg* gene, the alpha cells could synthesize more GLP-1 under these experimental conditions. We then measured GLP-1 content and secretion and found that cells chronically exposed to GLP-1 had higher intracellular GLP-1 content levels and secreted more GLP-1. Accordingly, we also observed an increase in PC1/3, the protein convertase that cleaves proglucagon to GLP-1; in contrast, levels of PC2, the protein convertase that cleaves proglucagon to glucagon, were unchanged. Although it is known that Pax6, in particular in pancreatic beta cells, could influences PC2 expression [51], in our model we did not found any difference in PC2 expression between control and treated groups. In our opinion this phenomenon occurs because in our model (alpha cells) PC2 is constitutively active and a further stimulation by Pax6 does not lead to a significant increase in PC2 expression. For this reason probably we cannot observe any variation in expression of PC2 after GLP-1 treatment.

Additionally, we cultured α-TC1-6 cells in the presence of Exendin-4 (a GLP-1 analogue) and observed similar GLP-1 secretion and content levels as in cells cultured in the presence of GLP-1. As a result, our data indicate that the prolonged exposure of pancreatic alpha cells to GLP-1 increases the ability of these cells to produce and secrete GLP-1.

Previous studies have shown that alpha cells (or at least a subpopulation of alpha cells) are able to release GLP-1. Fully processed GLP-1 was identified in rat pancreatic extracts with the use of chromatographic analysis and radioimmunoassays [52]. Immunoreactive GLP-1 was detected in alpha cells, and its release increased in response to stimulation with glucose [53]. Kreymann et al. detected GLP-1 (7–36) amide in the pancreas of fetal and neonatal rats [54]. Relevant amounts of fully processed GLP-1 were produced by primary alpha cells from isolated rat islets [55]. When partial beta cell loss was induced in neonatal rats by streptozotocin, islet cell regeneration was found to accompany the hyperplasia of alpha cells with an altered phenotype (specifically, increased GLP-1 synthesis) [56]. More recently, the processing of proglucagon to GLP-1 has been reported to occur in α-TC1-6 cells and in rat and human islets [57]. A recent study in rats showed that the cytokine interleukin-6 was able to increase GLP-1 release from L cells and alpha cells; some of these results have been reproduced with human islets [58]. Finally, a local GLP-1 system has been shown to be present in human pancreatic islets. The production of GLP-1 occurs in the alpha cells and is modulated by nutrients and affected by type 2 diabetes [15]. Other studies have confirmed that GLP-1 production may be modulated under certain conditions. For example, the release of GLP-1 from alpha cells was found to be up-regulated in *P. obesus* during the development of diabetes [59]. Additionally, a model of beta cell regeneration has been characterized by the hyperplasia of alpha cells with an increased capacity for GLP-1 synthesis.

In our model, we also found that the prolonged exposure of alpha cells to GLP-1 induced the expression of the *Pax6* gene and the production of the Pax6 protein and the protein convertase PC1/3. The transcription factor *Pax6* regulates the production of glucagon, insulin and somatostatin [60], [61]. *Pax6* has also been shown to control the expression of the *Gcg* gene through its binding to the G1 and G3 elements on the *Gcg* promoter [40]. The proglucagon protein contains multiple cleavage sites that are recognized with various degrees of efficiency by the pro-hormone convertases PC1/3 and PC2. In pancreatic alpha cells, the abundant expression of PC2 is almost exclusively associated with the production of glucagon. However, PC1/3 is also present in these cells and its expression can be modulated under various circumstances. For example, the adenovirus-mediated expression of PC1/3 in alpha cells increases the secretion of GLP-1 from islets, resulting in improved glucose-stimulated insulin secretion and enhanced survival in response to cytokine treatment [62]. Moreover, previous reports have demonstrated that the expression of PC1/3 rather than PC2 in alpha cells induces GLP-1 and GLP-2 production and promotes islet survival after transplantation [63]. The same authors also examined the metabolic effects of transplanting encapsulated PC1/3-expressing α-cells in rodent models of T2D and demonstrated that cell therapy with PC1/3- expressing α-cells was able to improve glucose handling [64].

These results appear to contradict the traditional paradigms of endocrine systems whereby “autocrine” signals usually generate negative feed-back on the same hormonal-producing cells to regulate hormonal production. Alpha cell hold the necessary machinery to produce GLP-1 and, although at low levels, we could detect GLP-1 release not only in our model (α-TC1-6) but also in isolated rat islets chronically exposed to Exendin-4. Although pancreatic alpha cells do not conventionally produce GLP-1, its detection in islets or in isolated alpha cells chronically cultured with GLP-1 (α-TC1-6) demonstrates a positive stimulatory effect of GLP-1 on its own synthesis. In agreement with our results, other authors studying pancreatic alpha cells have recently demonstrated that glucagon regulates its own synthesis via positive autocrine signalling [65]. It is possible that a similar regulation could apply to the action of GLP-1 on proglucagon, PC1/3 expression and GLP-1 production. Moreover, the same authors demonstrated that GLP-1 stimulated the activity of the glucagon-promoter in α-TC1-6 cells, perhaps due to a similar effect as that observed on *Gcg* gene expression in the present study.

In summary, our data indicate that the chronic exposure of pancreatic alpha cells to GLP-1 increases the ability of these cells to produce and release GLP-1. This phenomenon occurs through the stimulation of the transcription factor *Pax6* and an increase in the expression of the protein convertase PC1/3. Although it might be too speculative to draw definitive conclusions from an *in vitro* study, our findings suggest that these effects might also occur in patients chronically treated with GLP-1 analogues or DPP-4 inhibitors, thus contributing to the positive effects of these drugs on islet function during treatment.
